# 2,3-Bis(thio­phen-2-yl)pyrazine­[2,3-*f*][1,10]phenanthroline

**DOI:** 10.1107/S160053681201522X

**Published:** 2012-04-21

**Authors:** Chang-Ge Zheng, Jun Kong, Peng Zhang, Wen-Xian Dong

**Affiliations:** a School of Chemical and Material Engineering, Jiangnan University, 1800 Lihu Road, Wuxi, Jiangsu Province 214122, People’s Republic of China, and Key Laboratory of Organofluorine Chemistry, Shanghai Institute of Organic Chemistry, 345 Lingling Road, Shanghai 200032, People’s Republic of China

## Abstract

The mol­ecule of the title compound, C_22_H_12_N_4_S_2_, shows no crystallographic symmetry. The thiophene rings form different dihedral angles [40.15 (9) and 15.43 (10)°] with the pyrazine ring. A strong π–π stacking inter­action occurs between adjacent pyrazine­[2,3-*f*][1,10]phenanthroline units with an inter­planar distance of 3.4352 (16) Å.

## Related literature

For the structure of 2,3-dithienyl­pyrazine­[2,3-*f*]-1,10-phenanthroline, see: Chen & Li (2004 ▶). For the properties of 2,3-dithienyl­pyrazine­[2,3-*f*]-1,10-phenanthroline, see: Armaroli *et al.* (1992 ▶); Aragoni *et al.* (2002 ▶); Bencini *et al.* (1999 ▶).
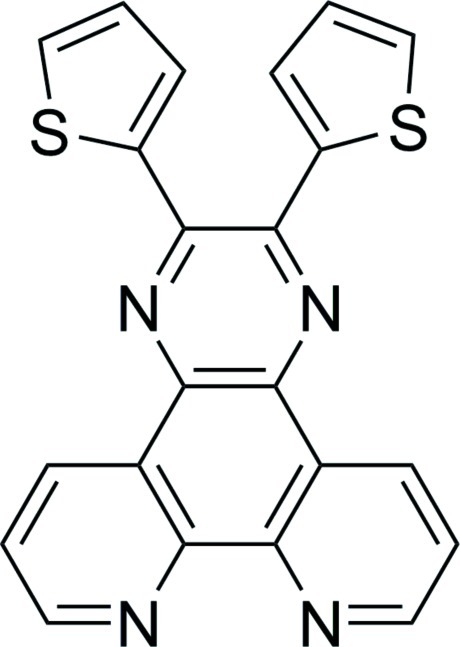


## Experimental

### 

#### Crystal data

C_22_H_12_N_4_S_2_
*M* *_r_* = 396.48
Monoclinic, 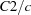

*a* = 27.016 (5) Å
*b* = 10.267 (2) Å
*c* = 13.835 (3) Å
β = 117.04 (3)°
*V* = 3418.1 (15) Å^3^
*Z* = 8
Mo *K*α radiation
μ = 0.33 mm^−1^
*T* = 293 K
0.30 × 0.30 × 0.10 mm

#### Data collection

Bruker SMART APEXII CCD area-detector diffractometer
Absorption correction: multi-scan (*SADABS*; Sheldrick, 1996 ▶) *T*_min_ = 0.763, *T*_max_ = 1.000
9577 measured reflections
3867 independent reflections
3057 reflections with *I* > 2σ(*I*)
*R*_int_ = 0.039

#### Refinement

*R*[*F* ^2^ > 2σ(*F* ^2^)] = 0.063
*wR*(*F* ^2^) = 0.130
*S* = 1.14
3867 reflections
253 parameters
H-atom parameters constrained
Δρ_max_ = 0.41 e Å^−3^
Δρ_min_ = −0.45 e Å^−3^

### 

Data collection: *APEX2* (Bruker, 2005 ▶); cell refinement: *SAINT* (Bruker, 2005 ▶); data reduction: *SAINT*; program(s) used to solve structure: *SHELXS97* (Sheldrick, 2008 ▶); program(s) used to refine structure: *SHELXL97* (Sheldrick, 2008 ▶); molecular graphics: *PLATON* (Spek, 2009 ▶); software used to prepare material for publication: *SHELXL97*.

## Supplementary Material

Crystal structure: contains datablock(s) global, I. DOI: 10.1107/S160053681201522X/aa2049sup1.cif

Structure factors: contains datablock(s) I. DOI: 10.1107/S160053681201522X/aa2049Isup2.hkl

Additional supplementary materials:  crystallographic information; 3D view; checkCIF report

## Figures and Tables

**Fig. 1.**
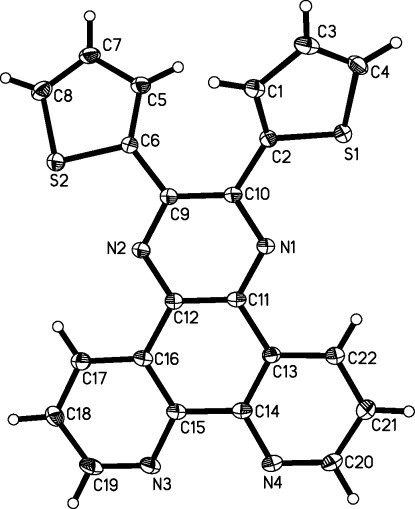
The structure of the title compound showing 30% probability displacement ellipsoids and the atom-numbering scheme.

## supplementary crystallographic information

### Comment

2,3-Dithienylpyrazine[2,3-*f*]-1,10-phenanthroline as a ligand is widely used as analytical probes, such as proton, ion sensors and organic light-emitting devices (Armaroli *et al.*, 1992; Aragoni *et al.*, 2002; Bencini *et al.*, 1999; Chen & Li, 2004), due to its rigid structure and fluorescence property.

The molecule of the title compound, C_22_H_12_N_4_S_2_, is chemically symmetric but it shows no crystallographic symmetry. The dihedral angles between thiophene rings and pyrazine ring are 40.15 (9)° and 15.43 (10)°, respectively. The strong π-π stacking occurs in the crystal structure between parallel pyrazine[2,3-*f*]-1,10-phenanthroline molecules, the interplanar distance is 3.4352 (16) Å.

### Experimental

The title compound was synthesized by using 1,10-phenanthroline as the starting material according to the published route (Chen & Li, 2004). The single crystals were obtained by recrystallization from the mixture of methanol and methylene chloride at room temperature.

### Refinement

All H atoms were placed in geometrically idealized positions and constrained to ride on their parent atoms with C—H distance of 0.93 Å, and with *U*_iso_(H)=1.2*U*_iso_(C).

### Crystal data

**Table:**

C_22_H_12_N_4_S_2_	*F*(000) = 1632
*M**_r_* = 396.48	*D*_x_ = 1.541 Mg m^−^^3^
Monoclinic, *C*2/*c*	Mo *K*α radiation, λ = 0.71073 Å
Hall symbol: -C 2yc	Cell parameters from 7427 reflections
*a* = 27.016 (5) Å	θ = 3.0–27.5°
*b* = 10.267 (2) Å	µ = 0.33 mm^−^^1^
*c* = 13.835 (3) Å	*T* = 293 K
β = 117.04 (3)°	Block, yellow
*V* = 3418.1 (15) Å^3^	0.30 × 0.30 × 0.10 mm
*Z* = 8	

### Data collection

**Table:**

Bruker SMART APEXII CCD area-detector diffractometer	3867 independent reflections
Radiation source: fine-focus sealed tube	3057 reflections with *I* > 2σ(*I*)
Graphite monochromator	*R*_int_ = 0.039
Detector resolution: 28.5714 pixels mm^-1^	θ_max_ = 27.5°, θ_min_ = 3.1°
phi and ω scans	*h* = −34→34
Absorption correction: multi-scan (*SADABS*; Sheldrick, 1996)	*k* = −13→11
*T*_min_ = 0.763, *T*_max_ = 1.000	*l* = −17→15
9577 measured reflections	

### Refinement

**Table:**

Refinement on *F*^2^	Primary atom site location: structure-invariant direct methods
Least-squares matrix: full	Secondary atom site location: difference Fourier map
*R*[*F*^2^ > 2σ(*F*^2^)] = 0.063	Hydrogen site location: inferred from neighbouring sites
*wR*(*F*^2^) = 0.130	H-atom parameters constrained
*S* = 1.14	*w* = 1/[σ^2^(*F*_o_^2^) + (0.0537*P*)^2^ + 1.3889*P*] where *P* = (*F*_o_^2^ + 2*F*_c_^2^)/3
3867 reflections	(Δ/σ)_max_ < 0.001
253 parameters	Δρ_max_ = 0.41 e Å^−^^3^
0 restraints	Δρ_min_ = −0.45 e Å^−^^3^

### Special details

**Table:**

Geometry. All e.s.d.'s (except the e.s.d. in the dihedral angle between two l.s. planes) are estimated using the full covariance matrix. The cell e.s.d.'s are taken into account individually in the estimation of e.s.d.'s in distances, angles and torsion angles; correlations between e.s.d.'s in cell parameters are only used when they are defined by crystal symmetry. An approximate (isotropic) treatment of cell e.s.d.'s is used for estimating e.s.d.'s involving l.s. planes.
Refinement. Refinement of *F*^2^ against ALL reflections. The weighted *R*-factor *wR* and goodness of fit *S* are based on *F*^2^, conventional *R*-factors *R* are based on *F*, with *F* set to zero for negative *F*^2^. The threshold expression of *F*^2^ > σ(*F*^2^) is used only for calculating *R*-factors(gt) *etc*. and is not relevant to the choice of reflections for refinement. *R*-factors based on *F*^2^ are statistically about twice as large as those based on *F*, and *R*- factors based on ALL data will be even larger.

### Fractional atomic coordinates and isotropic or equivalent isotropic displacement parameters (Å^2^)

**Table:**

	*x*	*y*	*z*	*U*_iso_*/*U*_eq_	
S1	0.20867 (3)	0.16082 (6)	0.37184 (6)	0.0384 (2)	
S2	0.10144 (3)	−0.41137 (6)	0.25638 (6)	0.0385 (2)	
C13	−0.00055 (10)	0.1677 (2)	0.12409 (19)	0.0261 (5)	
C16	−0.04190 (9)	−0.0932 (2)	0.07706 (18)	0.0253 (5)	
N4	−0.09519 (9)	0.2440 (2)	0.02141 (17)	0.0345 (5)	
C11	0.03747 (9)	0.0587 (2)	0.16463 (18)	0.0245 (5)	
C12	0.01743 (9)	−0.0683 (2)	0.14262 (18)	0.0245 (5)	
N1	0.09256 (8)	0.08207 (19)	0.21974 (15)	0.0259 (4)	
C9	0.10654 (9)	−0.1492 (2)	0.24045 (18)	0.0243 (5)	
N2	0.05183 (8)	−0.17051 (19)	0.18208 (15)	0.0260 (4)	
N3	−0.13437 (8)	−0.0055 (2)	−0.02387 (16)	0.0318 (5)	
C15	−0.07901 (9)	0.0118 (2)	0.03844 (18)	0.0264 (5)	
C14	−0.05811 (10)	0.1460 (2)	0.06220 (18)	0.0274 (5)	
C10	0.12755 (9)	−0.0183 (2)	0.25369 (18)	0.0244 (5)	
C18	−0.11870 (11)	−0.2366 (3)	−0.0142 (2)	0.0362 (6)	
H18	−0.1337	−0.3196	−0.0343	0.043*	
C19	−0.15243 (10)	−0.1264 (3)	−0.0490 (2)	0.0363 (6)	
H19	−0.1902	−0.1389	−0.0929	0.044*	
C2	0.18659 (10)	0.0169 (2)	0.30092 (19)	0.0267 (5)	
C6	0.13841 (10)	−0.2672 (2)	0.28959 (18)	0.0260 (5)	
C1	0.22833 (10)	−0.0375 (3)	0.28392 (19)	0.0307 (6)	
H1	0.2246	−0.1151	0.2465	0.037*	
C5	0.19175 (10)	−0.2883 (3)	0.36968 (19)	0.0312 (6)	
H5	0.2183	−0.2229	0.3993	0.037*	
C3	0.27773 (10)	0.0370 (3)	0.3294 (2)	0.0358 (6)	
H3	0.3099	0.0135	0.3251	0.043*	
C21	−0.01907 (11)	0.3958 (3)	0.0982 (2)	0.0426 (7)	
H21	−0.0073	0.4821	0.1076	0.051*	
C8	0.15717 (11)	−0.4972 (3)	0.3474 (2)	0.0394 (7)	
H8	0.1568	−0.5866	0.3581	0.047*	
C20	−0.07546 (11)	0.3638 (3)	0.0395 (2)	0.0390 (7)	
H20	−0.1007	0.4319	0.0113	0.047*	
C17	−0.06273 (10)	−0.2195 (3)	0.0506 (2)	0.0332 (6)	
H17	−0.0391	−0.2909	0.0764	0.040*	
C4	0.27329 (10)	0.1460 (3)	0.3795 (2)	0.0381 (7)	
H4	0.3019	0.2056	0.4140	0.046*	
C22	0.01805 (11)	0.2963 (2)	0.1410 (2)	0.0340 (6)	
H22	0.0557	0.3143	0.1815	0.041*	
C7	0.20172 (10)	−0.4207 (3)	0.4020 (2)	0.0344 (6)	
H7	0.2355	−0.4514	0.4553	0.041*	

### Atomic displacement parameters (Å^2^)

**Table:**

	*U*^11^	*U*^22^	*U*^33^	*U*^12^	*U*^13^	*U*^23^
S1	0.0269 (3)	0.0271 (3)	0.0511 (4)	−0.0011 (3)	0.0089 (3)	−0.0050 (3)
S2	0.0289 (4)	0.0258 (3)	0.0491 (4)	−0.0013 (3)	0.0075 (3)	0.0031 (3)
C13	0.0225 (12)	0.0267 (13)	0.0261 (12)	0.0035 (10)	0.0085 (10)	0.0005 (10)
C16	0.0202 (11)	0.0317 (13)	0.0231 (11)	0.0009 (10)	0.0089 (9)	0.0029 (10)
N4	0.0266 (11)	0.0325 (12)	0.0386 (12)	0.0080 (9)	0.0096 (10)	0.0036 (10)
C11	0.0197 (11)	0.0297 (13)	0.0236 (12)	0.0034 (10)	0.0093 (9)	0.0021 (10)
C12	0.0225 (12)	0.0267 (12)	0.0242 (11)	0.0015 (10)	0.0105 (9)	0.0012 (10)
N1	0.0210 (10)	0.0258 (10)	0.0271 (10)	0.0020 (8)	0.0077 (8)	0.0001 (9)
C9	0.0223 (12)	0.0258 (12)	0.0238 (11)	0.0011 (9)	0.0096 (9)	0.0005 (10)
N2	0.0212 (10)	0.0274 (11)	0.0262 (10)	0.0008 (8)	0.0079 (8)	0.0010 (9)
N3	0.0218 (10)	0.0381 (12)	0.0305 (11)	−0.0004 (9)	0.0074 (9)	0.0013 (10)
C15	0.0211 (12)	0.0328 (13)	0.0243 (12)	0.0022 (10)	0.0092 (10)	0.0044 (10)
C14	0.0230 (12)	0.0335 (13)	0.0251 (12)	0.0046 (10)	0.0106 (10)	0.0016 (11)
C10	0.0210 (12)	0.0264 (12)	0.0233 (12)	0.0029 (10)	0.0077 (9)	0.0020 (10)
C18	0.0293 (14)	0.0332 (14)	0.0412 (15)	−0.0082 (11)	0.0117 (12)	−0.0007 (12)
C19	0.0182 (12)	0.0503 (17)	0.0353 (14)	−0.0029 (12)	0.0077 (10)	0.0054 (13)
C2	0.0237 (12)	0.0233 (12)	0.0279 (12)	−0.0011 (9)	0.0071 (10)	0.0027 (10)
C6	0.0221 (12)	0.0245 (12)	0.0284 (12)	0.0001 (9)	0.0089 (10)	−0.0002 (10)
C1	0.0243 (12)	0.0342 (14)	0.0315 (13)	0.0010 (11)	0.0108 (10)	0.0010 (11)
C5	0.0257 (13)	0.0311 (14)	0.0311 (13)	0.0022 (10)	0.0080 (10)	0.0045 (11)
C3	0.0226 (13)	0.0481 (17)	0.0348 (14)	0.0024 (12)	0.0113 (11)	0.0099 (13)
C21	0.0339 (15)	0.0288 (14)	0.0552 (18)	0.0039 (11)	0.0117 (13)	−0.0015 (13)
C8	0.0373 (15)	0.0282 (14)	0.0492 (17)	0.0078 (12)	0.0167 (13)	0.0104 (13)
C20	0.0330 (14)	0.0303 (14)	0.0443 (16)	0.0113 (12)	0.0094 (12)	0.0040 (12)
C17	0.0268 (13)	0.0314 (14)	0.0368 (14)	0.0008 (11)	0.0104 (11)	0.0034 (12)
C4	0.0225 (13)	0.0377 (15)	0.0418 (15)	−0.0073 (11)	0.0041 (11)	0.0071 (13)
C22	0.0247 (13)	0.0293 (13)	0.0417 (15)	0.0030 (10)	0.0097 (11)	−0.0014 (12)
C7	0.0265 (13)	0.0337 (14)	0.0363 (14)	0.0067 (11)	0.0083 (11)	0.0085 (12)

### Geometric parameters (Å, º)

**Table:**

S1—C4	1.707 (3)	C18—C17	1.374 (3)
S1—C2	1.723 (2)	C18—C19	1.394 (4)
S2—C8	1.704 (3)	C18—H18	0.9300
S2—C6	1.727 (2)	C19—H19	0.9300
C13—C22	1.395 (3)	C2—C1	1.371 (3)
C13—C14	1.411 (3)	C6—C5	1.378 (3)
C13—C11	1.449 (3)	C1—C3	1.414 (3)
C16—C17	1.394 (3)	C1—H1	0.9300
C16—C15	1.402 (3)	C5—C7	1.418 (4)
C16—C12	1.462 (3)	C5—H5	0.9300
N4—C20	1.318 (3)	C3—C4	1.351 (4)
N4—C14	1.349 (3)	C3—H3	0.9300
C11—N1	1.350 (3)	C21—C22	1.364 (4)
C11—C12	1.391 (3)	C21—C20	1.401 (4)
C12—N2	1.343 (3)	C21—H21	0.9300
N1—C10	1.331 (3)	C8—C7	1.345 (4)
C9—N2	1.342 (3)	C8—H8	0.9300
C9—C10	1.437 (3)	C20—H20	0.9300
C9—C6	1.463 (3)	C17—H17	0.9300
N3—C19	1.321 (3)	C4—H4	0.9300
N3—C15	1.356 (3)	C22—H22	0.9300
C15—C14	1.468 (3)	C7—H7	0.9300
C10—C2	1.467 (3)		
			
C4—S1—C2	92.22 (13)	C1—C2—C10	129.5 (2)
C8—S2—C6	92.10 (13)	C1—C2—S1	110.35 (18)
C22—C13—C14	117.7 (2)	C10—C2—S1	119.32 (18)
C22—C13—C11	121.9 (2)	C5—C6—C9	133.1 (2)
C14—C13—C11	120.3 (2)	C5—C6—S2	110.26 (19)
C17—C16—C15	118.8 (2)	C9—C6—S2	116.07 (17)
C17—C16—C12	121.6 (2)	C2—C1—C3	112.8 (2)
C15—C16—C12	119.6 (2)	C2—C1—H1	123.6
C20—N4—C14	117.2 (2)	C3—C1—H1	123.6
N1—C11—C12	120.7 (2)	C6—C5—C7	112.5 (2)
N1—C11—C13	119.1 (2)	C6—C5—H5	123.7
C12—C11—C13	120.2 (2)	C7—C5—H5	123.7
N2—C12—C11	121.0 (2)	C4—C3—C1	113.0 (2)
N2—C12—C16	118.4 (2)	C4—C3—H3	123.5
C11—C12—C16	120.5 (2)	C1—C3—H3	123.5
C10—N1—C11	119.0 (2)	C22—C21—C20	117.9 (3)
N2—C9—C10	119.5 (2)	C22—C21—H21	121.1
N2—C9—C6	113.5 (2)	C20—C21—H21	121.1
C10—C9—C6	126.9 (2)	C7—C8—S2	112.2 (2)
C9—N2—C12	119.1 (2)	C7—C8—H8	123.9
C19—N3—C15	117.3 (2)	S2—C8—H8	123.9
N3—C15—C16	122.1 (2)	N4—C20—C21	124.6 (2)
N3—C15—C14	117.8 (2)	N4—C20—H20	117.7
C16—C15—C14	120.0 (2)	C21—C20—H20	117.7
N4—C14—C13	122.7 (2)	C18—C17—C16	118.9 (2)
N4—C14—C15	118.0 (2)	C18—C17—H17	120.5
C13—C14—C15	119.3 (2)	C16—C17—H17	120.5
N1—C10—C9	120.2 (2)	C3—C4—S1	111.7 (2)
N1—C10—C2	114.8 (2)	C3—C4—H4	124.2
C9—C10—C2	125.0 (2)	S1—C4—H4	124.2
C17—C18—C19	118.3 (2)	C21—C22—C13	119.9 (2)
C17—C18—H18	120.9	C21—C22—H22	120.0
C19—C18—H18	120.9	C13—C22—H22	120.0
N3—C19—C18	124.5 (2)	C8—C7—C5	112.9 (2)
N3—C19—H19	117.7	C8—C7—H7	123.5
C18—C19—H19	117.7	C5—C7—H7	123.5
